# Supplementary material for: A phase 2 single center open label randomised control trial for convalescent plasma therapy in patients with severe COVID-19
Source: Nat Commun. 2022 Jan 19;13:383. doi: 10.1038/s41467-022-28064-7 (PMC8770561; doi:10.1038/s41467-022-28064-7)
Supplement: Supplementary file 1 — Supplementary Information [file 41467_2022_28064_MOESM1_ESM.pdf]

A phase 2 single center open label randomised control trial for convalescent plasma therapy in patients with severe COVID-19

Yogiraj Ray et al.

Supplemental information

Legends to supplemental figures

Supplemental figures 1-3

Supplemental tables 1-4

Legends to supplemental figures

Supplemental figure 1. Additional characterization of convalescent donors. (A) Day since being tested positive for SARS-CoV2 on RT-PCR for all convalescent donors. **(B)** Correlation between anti-spike IgG content of CP and day since the donors were positive on RT-PCR. **(C)** Distribution of the donor cohort for WHO Clinical Progression Score for their disease courses are plotted against the anti-spike IgG content of CP. Pearson correlation was computed.

Supplemental figure 2. Comparison between patient characteristics between two trial arms. (A) Comparison of age distribution between patients randomize to SOC and CPT arms. **(B)** Comparison between viral load on the day of enrolment between patients randomize to SOC and CPT arms. **(C)** Comparison between anti-SARS-CoV-2 neutralizing antibody activity of plasma on the day of enrolment between patients randomized to SOC and CPT arms. **(D)** Comparison between SpO2/FiO2 ratio on the day of enrolment between patients randomized to SOC and CPT arms. **(E)** Comparison between the kinetics of respiratory rate from the day before the enrolment day till 10th day post-enrolment between patients randomized to SOC and CPT arms. **(F)** Comparison between the kinetics of heart rate from the day before the enrolment day till 10th day post-enrolment between patients randomized to SOC and CPT arms. **(G)** Kinetics of systolic and diastolic blood pressure from the day before the enrolment day till 10th day post-enrolment in patients randomized to SOC arm. **(H)** Kinetics of systolic and diastolic blood pressure from the day before the enrolment day till 10th day post-enrolment in patients randomized to CPT arm.

Supplemental figure 3. Distribution of cytokine expression levels in the patient cohort. Density histogram of plasma abundance levels of 36 cytokines at T1 and T2 time points. Upper panel represents the change in population distribution of cytokine abundance for SOC arm from T1 to T2 time point and lower panel represents the same for CPT arm. The pink and blue colour indicates the distribution profiles of indicated cytokines at T1 and T2 time points respectively. The red stars are marking significant differences in median values between T1 and T2.

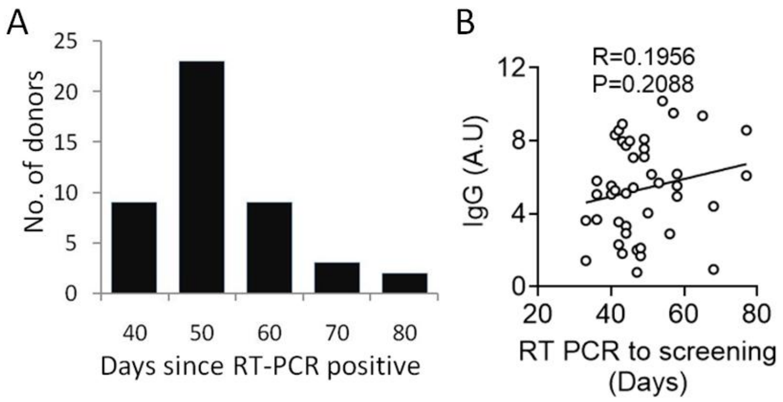

Supplemental figure 1

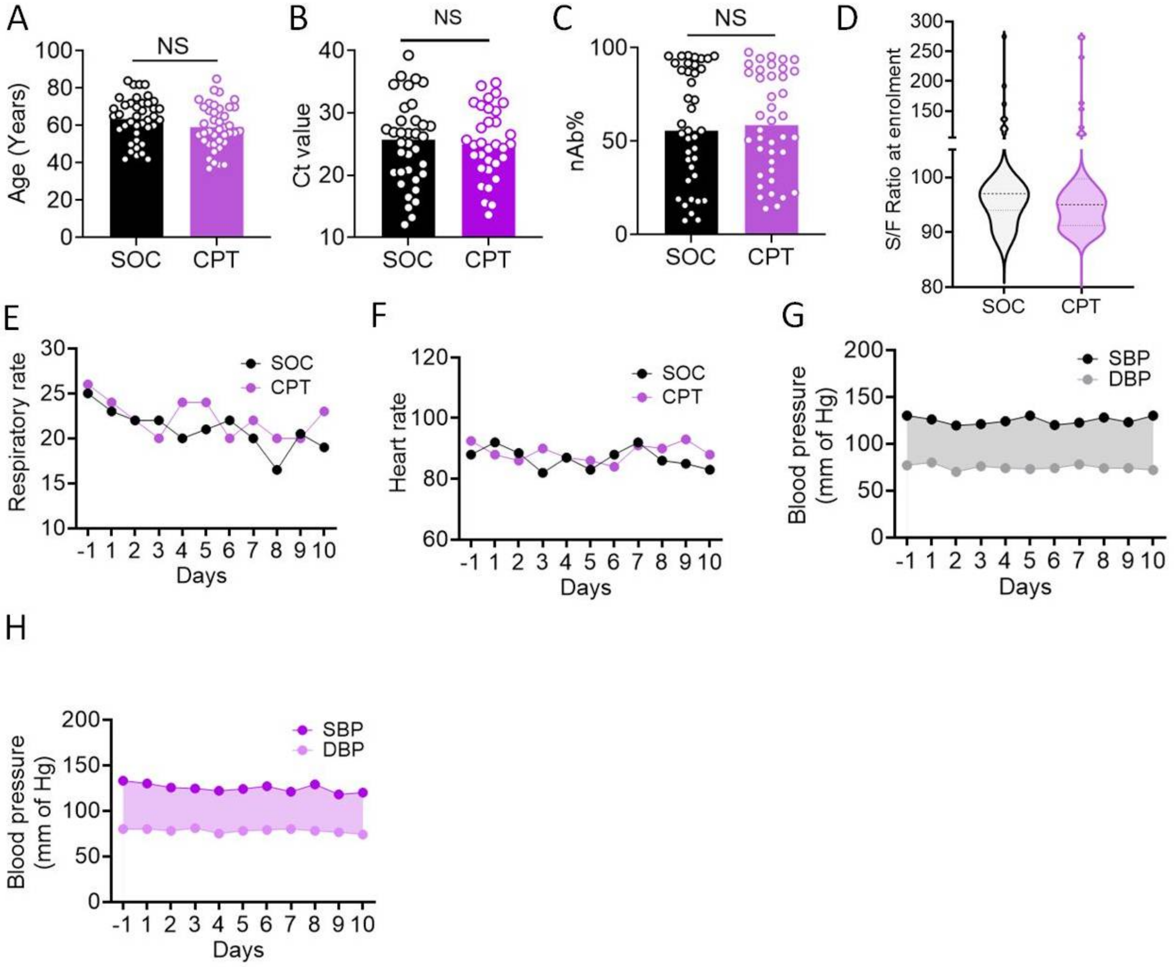

Supplemental figure 2

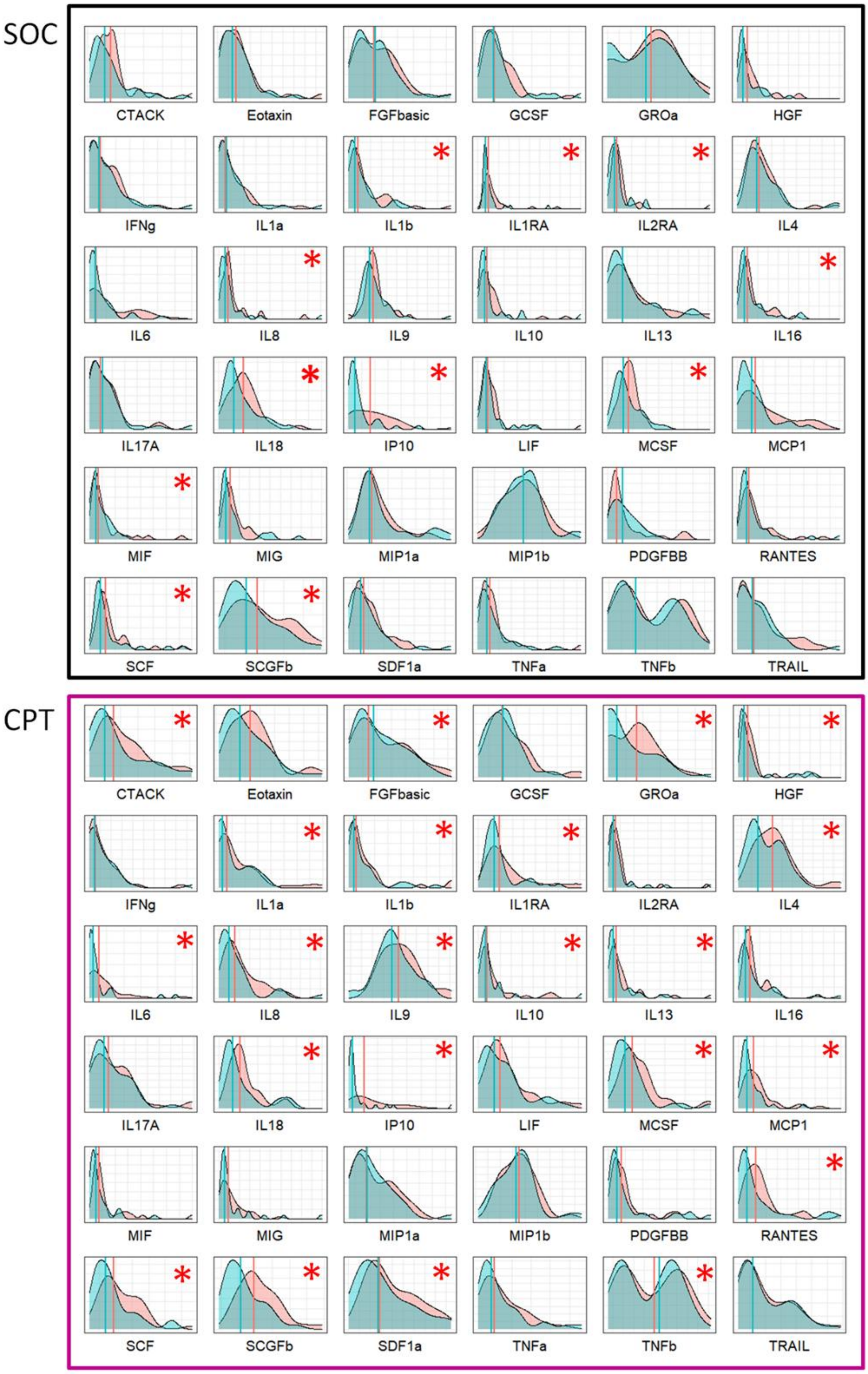

Supplemental figure 3

Supplemental table 1. List of proteins identified in the proteomics experiment on convalescent plasma.

Index	Peak Name	Group
1	sp|P02768|ALBU_HUMAN	Serum albumin OS=Homo sapiens OX=9606 GN=ALB PE=1 SV=2
2	sp|P04114|APOB_HUMAN	Apolipoprotein B-100 OS=Homo sapiens OX=9606 GN=APOB PE=1 SV=2
3	sp|P01024|CO3_HUMAN	Complement C3 OS=Homo sapiens OX=9606 GN=C3 PE=1 SV=2
4	sp|P01023|A2MG_HUMAN	Alpha-2-macroglobulin OS=Homo sapiens OX=9606 GN=A2M PE=1 SV=3
5	sp|P02787|TRFE_HUMAN	Serotransferrin OS=Homo sapiens OX=9606 GN=TF PE=1 SV=3
6	sp|P0C0L4|CO4A_HUMAN	Complement C4-A OS=Homo sapiens OX=9606 GN=C4A PE=1 SV=2
7	sp|P00450|CERU_HUMAN	Ceruloplasmin OS=Homo sapiens OX=9606 GN=CP PE=1 SV=1
8	sp|P01009|A1AT_HUMAN	Alpha-1-antitrypsin OS=Homo sapiens OX=9606 GN=SERPINA1 PE=1 SV=3
9	tr|A0A0A0MS08|A0A0A0MS08_HUMAN	Immunoglobulin heavy constant gamma 1 (Fragment) OS=Homo sapiens OX=9606 GN=IGHG1 PE=1 SV=1
10	sp|P02751|FINC_HUMAN	Fibronectin OS=Homo sapiens OX=9606 GN=FN1 PE=1 SV=4
11	sp|P02671|FIBA_HUMAN	Fibrinogen alpha chain OS=Homo sapiens OX=9606 GN=FGA PE=1 SV=2
12	sp|P08603|CFAH_HUMAN	Complement factor H OS=Homo sapiens OX=9606 GN=CFH PE=1 SV=4
13	sp|P02675|FIBB_HUMAN	Fibrinogen beta chain OS=Homo sapiens OX=9606 GN=FGB PE=1 SV=2
14	sp|P00738|HPT_HUMAN	Haptoglobin OS=Homo sapiens OX=9606 GN=HP PE=1 SV=1
15	tr|B4E1Z4|B4E1Z4_HUMAN	cDNA FLJ55673, highly similar to Complement factor B OS=Homo sapiens OX=9606 PE=1 SV=1
16	sp|P02679|FIBG_HUMAN	Fibrinogen gamma chain OS=Homo sapiens OX=9606 GN=FGG PE=1 SV=3
17	sp|P00747|PLMN_HUMAN	Plasminogen OS=Homo sapiens OX=9606 GN=PLG PE=1 SV=2
18	tr|B7ZKJ8|B7ZKJ8_HUMAN	ITIH4 protein OS=Homo sapiens OX=9606 GN=ITIH4 PE=1 SV=1
19	sp|P02774|VTDB_HUMAN	Vitamin D-binding protein OS=Homo sapiens OX=9606 GN=GC PE=1 SV=2
20	sp|P02647|APOA1_HUMAN	Apolipoprotein A-I OS=Homo sapiens OX=9606 GN=APOA1 PE=1 SV=1
21	sp|P01031|CO5_HUMAN	Complement C5 OS=Homo sapiens OX=9606 GN=C5 PE=1 SV=4
22	sp|P00734|THRB_HUMAN	Prothrombin OS=Homo sapiens OX=9606 GN=F2 PE=1 SV=2
23	tr|Q5T985|Q5T985_HUMAN	Inter-alpha-trypsin inhibitor heavy chain H2 OS=Homo sapiens OX=9606 GN=ITIH2 PE=1 SV=1
24	sp|P01871|IGHM_HUMAN	Immunoglobulin heavy constant mu OS=Homo sapiens OX=9606 GN=IGHM PE=1 SV=4
25	sp|P02790|HEMO_HUMAN	Hemopexin OS=Homo sapiens OX=9606 GN=HPX PE=1 SV=2
26	sp|P06727|APOA4_HUMAN	Apolipoprotein A-IV OS=Homo sapiens OX=9606 GN=APOA4 PE=1 SV=3
27	sp|P01876|IGHA1_HUMAN	Immunoglobulin heavy constant alpha 1 OS=Homo sapiens OX=9606 GN=IGHA1 PE=1 SV=2
28	sp|P04003|C4BPA_HUMAN	C4b-binding protein alpha chain OS=Homo sapiens OX=9606 GN=C4BPA PE=1 SV=2
29	sp|P01008|ANT3_HUMAN	Antithrombin-III OS=Homo sapiens OX=9606 GN=SERPINC1 PE=1 SV=1
30	sp|P01011|AACT_HUMAN	Alpha-1-antichymotrypsin OS=Homo sapiens OX=9606 GN=SERPINA3 PE=1 SV=2
31	sp|P68871|HBB_HUMAN	Hemoglobin subunit beta OS=Homo sapiens OX=9606 GN=HBB PE=1 SV=2
32	sp|P19827|ITIH1_HUMAN	Inter-alpha-trypsin inhibitor heavy chain H1 OS=Homo sapiens OX=9606 GN=ITIH1 PE=1 SV=3

33	sp|P10643|CO7_HUMAN	Complement component C7 OS=Homo sapiens OX=9606 GN=C7 PE=1 SV=2
34	sp|P06396|GELS_HUMAN	Gelsolin OS=Homo sapiens OX=9606 GN=GSN PE=1 SV=1
35	sp|P02765|FETUA_HUMAN	Alpha-2-HS-glycoprotein OS=Homo sapiens OX=9606 GN=AHSG PE=1 SV=2
36	sp|P01042|KNG1_HUMAN	Kininogen-1 OS=Homo sapiens OX=9606 GN=KNG1 PE=1 SV=2
37	sp|P04217|A1BG_HUMAN	Alpha-1B-glycoprotein OS=Homo sapiens OX=9606 GN=A1BG PE=1 SV=4
38	sp|P02749|APOH_HUMAN	Beta-2-glycoprotein 1 OS=Homo sapiens OX=9606 GN=APOH PE=1 SV=3
39	sp|P02649|APOE_HUMAN	Apolipoprotein E OS=Homo sapiens OX=9606 GN=APOE PE=1 SV=1
40	sp|P05155|IC1_HUMAN	Plasma protease C1 inhibitor OS=Homo sapiens OX=9606 GN=SERPING1 PE=1 SV=2
41	sp|P43652|AFAM_HUMAN	Afamin OS=Homo sapiens OX=9606 GN=AFM PE=1 SV=1
42	sp|P13671|CO6_HUMAN	Complement component C6 OS=Homo sapiens OX=9606 GN=C6 PE=1 SV=3
43	sp|P10909|CLUS_HUMAN	Clusterin OS=Homo sapiens OX=9606 GN=CLU PE=1 SV=1
44	tr|A0A0B4J231|A0A0B4J231_HUMAN	Immunoglobulin lambda-like polypeptide 5 OS=Homo sapiens OX=9606 GN=IGLL5 PE=1 SV=1
45	sp|P02763|A1AG1_HUMAN	Alpha-1-acid glycoprotein 1 OS=Homo sapiens OX=9606 GN=ORM1 PE=1 SV=1
46	tr|V9GYM3|V9GYM3_HUMAN	Apolipoprotein A-II OS=Homo sapiens OX=9606 GN=APOA2 PE=1 SV=1
47	sp|O75882|ATRN_HUMAN	Attractin OS=Homo sapiens OX=9606 GN=ATRN PE=1 SV=2
48	sp|P25311|ZA2G_HUMAN	Zinc-alpha-2-glycoprotein OS=Homo sapiens OX=9606 GN=AZGP1 PE=1 SV=2
49	sp|P04196|HRG_HUMAN	Histidine-rich glycoprotein OS=Homo sapiens OX=9606 GN=HRG PE=1 SV=1
50	sp|P02760|AMBP_HUMAN	Protein AMBP OS=Homo sapiens OX=9606 GN=AMBP PE=1 SV=1
51	sp|Q96PD5|PGRP2_HUMAN	N-acetylmuramoyl-L-alanine amidase OS=Homo sapiens OX=9606 GN=PGLYRP2 PE=1 SV=1
52	sp|P01834|IGKC_HUMAN	Immunoglobulin kappa constant OS=Homo sapiens OX=9606 GN=IGKC PE=1 SV=2
53	sp|P08697|A2AP_HUMAN	Alpha-2-antiplasmin OS=Homo sapiens OX=9606 GN=SERPINF2 PE=1 SV=3
54	sp|P20742|PZP_HUMAN	Pregnancy zone protein OS=Homo sapiens OX=9606 GN=PZP PE=1 SV=4
55	tr|A0A286YEY4|A0A286YEY4_HUMAN	Immunoglobulin heavy constant gamma 2 (Fragment) OS=Homo sapiens OX=9606 GN=IGHG2 PE=1 SV=1
56	tr|B4DPQ0|B4DPQ0_HUMAN	Complement C1r subcomponent OS=Homo sapiens OX=9606 GN=C1R PE=1 SV=1
57	sp|P09871|C1S_HUMAN	Complement C1s subcomponent OS=Homo sapiens OX=9606 GN=C1S PE=1 SV=1
58	sp|P02748|CO9_HUMAN	Complement component C9 OS=Homo sapiens OX=9606 GN=C9 PE=1 SV=2
59	sp|P01019|ANGT_HUMAN	Angiotensinogen OS=Homo sapiens OX=9606 GN=AGT PE=1 SV=1
60	sp|P02766|TTHY_HUMAN	Transthyretin OS=Homo sapiens OX=9606 GN=TTR PE=1 SV=1
61	tr|G3XAM2|G3XAM2_HUMAN	Complement factor I OS=Homo sapiens OX=9606 GN=CFI PE=1 SV=1
62	sp|P02750|A2GL_HUMAN	Leucine-rich alpha-2-glycoprotein OS=Homo sapiens OX=9606 GN=LRG1 PE=1 SV=2
63	sp|P60709|ACTB_HUMAN	Actin, cytoplasmic 1 OS=Homo sapiens OX=9606 GN=ACTB PE=1 SV=1
64	sp|P01880|IGHD_HUMAN	Immunoglobulin heavy constant delta OS=Homo sapiens OX=9606 GN=IGHD PE=1 SV=3
65	sp|O43866|CD5L_HUMAN	CD5 antigen-like OS=Homo sapiens OX=9606 GN=CD5L PE=1 SV=1
66	tr|A0A087WW43|A0A087WW43_HUMAN	Inter-alpha-trypsin inhibitor heavy chain H3 OS=Homo sapiens OX=9606 GN=ITIH3 PE=1 SV=1

67	sp|P27169|PON1_HUMAN	Serum paraoxonase/arylesterase 1 OS=Homo sapiens OX=9606 GN=PON1 PE=1 SV=3
68	sp|P04004|VTNC_HUMAN	Vitronectin OS=Homo sapiens OX=9606 GN=VTN PE=1 SV=1
69	sp|Q08380|LG3BP_HUMAN	Galectin-3-binding protein OS=Homo sapiens OX=9606 GN=LGALS3BP PE=1 SV=1
70	sp|P36955|PEDF_HUMAN	Pigment epithelium-derived factor OS=Homo sapiens OX=9606 GN=SERPINF1 PE=1 SV=4
71	sp|P08185|CBG_HUMAN	Corticosteroid-binding globulin OS=Homo sapiens OX=9606 GN=SERPINA6 PE=1 SV=1
72	tr|H0YAC1|H0YAC1_HUMAN	Plasma kallikrein (Fragment) OS=Homo sapiens OX=9606 GN=KLKB1 PE=1 SV=1
73	sp|P05543|THBG_HUMAN	Thyroxine-binding globulin OS=Homo sapiens OX=9606 GN=SERPINA7 PE=1 SV=2
74	tr|A0A3B3ISJ1|A0A3B3ISJ1_HUMAN	Vitamin K-dependent protein S OS=Homo sapiens OX=9606 GN=PROS1 PE=1 SV=1
75	sp|P51884|LUM_HUMAN	Lumican OS=Homo sapiens OX=9606 GN=LUM PE=1 SV=2
76	tr|Q5VY30|Q5VY30_HUMAN	Retinol-binding protein OS=Homo sapiens OX=9606 GN=RBP4 PE=1 SV=2
77	sp|P22792|CPN2_HUMAN	Carboxypeptidase N subunit 2 OS=Homo sapiens OX=9606 GN=CPN2 PE=1 SV=3
78	tr|C9JF17|C9JF17_HUMAN	Apolipoprotein D (Fragment) OS=Homo sapiens OX=9606 GN=APOD PE=1 SV=1
79	sp|P35858|ALS_HUMAN	Insulin-like growth factor-binding protein complex acid labile subunit OS=Homo sapiens OX=9606 GN=IGFALS PE=1 SV=1
80	sp|P05546|HEP2_HUMAN	Heparin cofactor 2 OS=Homo sapiens OX=9606 GN=SERPIND1 PE=1 SV=3
81	tr|A0A286YES1|A0A286YES1_HUMAN	Immunoglobulin heavy constant gamma 3 (Fragment) OS=Homo sapiens OX=9606 GN=IGHG3 PE=1 SV=1
82	sp|P80108|PHLD_HUMAN	Phosphatidylinositol-glycan-specific phospholipase D OS=Homo sapiens OX=9606 GN=GPLD1 PE=1 SV=3
83	tr|J3KRP0|J3KRP0_HUMAN	Beta-Ala-His dipeptidase OS=Homo sapiens OX=9606 GN=CNDP1 PE=1 SV=2
84	sp|P00739|HPTR_HUMAN	Haptoglobin-related protein OS=Homo sapiens OX=9606 GN=HPR PE=2 SV=2
85	sp|P19652|A1AG2_HUMAN	Alpha-1-acid glycoprotein 2 OS=Homo sapiens OX=9606 GN=ORM2 PE=1 SV=2
86	tr|B0YIW2|B0YIW2_HUMAN	Apolipoprotein C-III OS=Homo sapiens OX=9606 GN=APOC3 PE=1 SV=1
87	tr|F5H7G1|F5H7G1_HUMAN	Complement component C8 beta chain OS=Homo sapiens OX=9606 GN=C8B PE=1 SV=1
88	sp|P69905|HBA_HUMAN	Hemoglobin subunit alpha OS=Homo sapiens OX=9606 GN=HBA1 PE=1 SV=2
89	sp|P07360|CO8G_HUMAN	Complement component C8 gamma chain OS=Homo sapiens OX=9606 GN=C8G PE=1 SV=3
90	sp|P05160|F13B_HUMAN	Coagulation factor XIII B chain OS=Homo sapiens OX=9606 GN=F13B PE=1 SV=3
91	sp|P07357|CO8A_HUMAN	Complement component C8 alpha chain OS=Homo sapiens OX=9606 GN=C8A PE=1 SV=2
92	sp|P01591|IGJ_HUMAN	Immunoglobulin J chain OS=Homo sapiens OX=9606 GN=JCHAIN PE=1 SV=4
93	tr|A0A286YFJ8|A0A286YFJ8_HUMAN	Immunoglobulin heavy constant gamma 4 (Fragment) OS=Homo sapiens OX=9606 GN=IGHG4 PE=1 SV=1
94	tr|E9PHK0|E9PHK0_HUMAN	Tetranectin OS=Homo sapiens OX=9606 GN=CLEC3B PE=1 SV=1
95	sp|P80748|LV321_HUMAN	Immunoglobulin lambda variable 3-21 OS=Homo sapiens OX=9606 GN=IGLV3-21 PE=1 SV=2
96	tr|A0A096LPE2|A0A096LPE2_HUMAN	SAA2-SAA4 readthrough OS=Homo sapiens OX=9606 GN=SAA2-SAA4 PE=4 SV=1
97	sp|P04264|K2C1_HUMAN	Keratin, type II cytoskeletal 1 OS=Homo sapiens OX=9606 GN=KRT1 PE=1 SV=6
98	tr|K7ER74|K7ER74_HUMAN	APOC4-APOC2 readthrough (NMD candidate) OS=Homo sapiens OX=9606 GN=APOC4-APOC2 PE=1 SV=1

99	sp|A0A0B4J1Y9|HV372_HUMAN	Immunoglobulin heavy variable 3-72 OS=Homo sapiens OX=9606 GN=IGHV3-72 PE=3 SV=1
100	sp|P43251|BTD_HUMAN	Biotinidase OS=Homo sapiens OX=9606 GN=BTD PE=1 SV=2
101	tr|A0A0J9YY99|A0A0J9YY99_HUMAN	Uncharacterized protein (Fragment) OS=Homo sapiens OX=9606 PE=1 SV=1
102	sp|P29622|KAIN_HUMAN	Kallistatin OS=Homo sapiens OX=9606 GN=SERPINA4 PE=1 SV=3
103	sp|O75636|FCN3_HUMAN	Ficolin-3 OS=Homo sapiens OX=9606 GN=FCN3 PE=1 SV=2
104	sp|O14791|APOL1_HUMAN	Apolipoprotein L1 OS=Homo sapiens OX=9606 GN=APOL1 PE=1 SV=5
105	sp|P04275|VWF_HUMAN	von Willebrand factor OS=Homo sapiens OX=9606 GN=VWF PE=1 SV=4
106	sp|P01619|KV320_HUMAN	Immunoglobulin kappa variable 3-20 OS=Homo sapiens OX=9606 GN=IGKV3-20 PE=1 SV=2
107	sp|P01599|KV117_HUMAN	Immunoglobulin kappa variable 1-17 OS=Homo sapiens OX=9606 GN=IGKV1-17 PE=1 SV=2
108	sp|P23083|HV102_HUMAN	Immunoglobulin heavy variable 1-2 OS=Homo sapiens OX=9606 GN=IGHV1-2 PE=1 SV=2
109	sp|P02747|C1QC_HUMAN	Complement C1q subcomponent subunit C OS=Homo sapiens OX=9606 GN=C1QC PE=1 SV=3
110	sp|P00915|CAH1_HUMAN	Carbonic anhydrase 1 OS=Homo sapiens OX=9606 GN=CA1 PE=1 SV=2
111	sp|P02042|HBD_HUMAN	Hemoglobin subunit delta OS=Homo sapiens OX=9606 GN=HBD PE=1 SV=2
112	sp|P00748|FA12_HUMAN	Coagulation factor XII OS=Homo sapiens OX=9606 GN=F12 PE=1 SV=3
113	tr|I3L145|I3L145_HUMAN	Sex hormone-binding globulin OS=Homo sapiens OX=9606 GN=SHBG PE=1 SV=1
114	sp|P01714|LV319_HUMAN	Immunoglobulin lambda variable 3-19 OS=Homo sapiens OX=9606 GN=IGLV3-19 PE=1 SV=2
115	sp|P01782|HV309_HUMAN	Immunoglobulin heavy variable 3-9 OS=Homo sapiens OX=9606 GN=IGHV3-9 PE=1 SV=2
116	sp|P0DOY2|IGLC2_HUMAN	Immunoglobulin lambda constant 2 OS=Homo sapiens OX=9606 GN=IGLC2 PE=1 SV=1
117	tr|H9KV75|H9KV75_HUMAN	Alpha-actinin-1 OS=Homo sapiens OX=9606 GN=ACTN1 PE=1 SV=1
118	sp|P02743|SAMP_HUMAN	Serum amyloid P-component OS=Homo sapiens OX=9606 GN=APCS PE=1 SV=2
119	tr|K7ERI9|K7ERI9_HUMAN	Apolipoprotein C-I (Fragment) OS=Homo sapiens OX=9606 GN=APOC1 PE=1 SV=1
120	sp|O95445|APOM_HUMAN	Apolipoprotein M OS=Homo sapiens OX=9606 GN=APOM PE=1 SV=2
121	sp|P00488|F13A_HUMAN	Coagulation factor XIII A chain OS=Homo sapiens OX=9606 GN=F13A1 PE=1 SV=4
122	sp|P01624|KV315_HUMAN	Immunoglobulin kappa variable 3-15 OS=Homo sapiens OX=9606 GN=IGKV3-15 PE=1 SV=2
123	sp|P04180|LCAT_HUMAN	Phosphatidylcholine-sterol acyltransferase OS=Homo sapiens OX=9606 GN=LCAT PE=1 SV=1
124	sp|A0A0C4DH38|HV551_HUMAN	Immunoglobulin heavy variable 5-51 OS=Homo sapiens OX=9606 GN=IGHV5-51 PE=3 SV=1
125	sp|P20851|C4BPB_HUMAN	C4b-binding protein beta chain OS=Homo sapiens OX=9606 GN=C4BPB PE=1 SV=1
126	sp|A0A0A0MS15|HV349_HUMAN	Immunoglobulin heavy variable 3-49 OS=Homo sapiens OX=9606 GN=IGHV3-49 PE=3 SV=1
127	sp|P00742|FA10_HUMAN	Coagulation factor X OS=Homo sapiens OX=9606 GN=F10 PE=1 SV=2
128	tr|D6R934|D6R934_HUMAN	Complement C1q subcomponent subunit B OS=Homo sapiens OX=9606 GN=C1QB PE=1 SV=1
129	sp|Q9NZP8|C1RL_HUMAN	Complement C1r subcomponent-like protein OS=Homo sapiens OX=9606 GN=C1RL PE=1 SV=2
130	tr|A0A087X0Q4|A0A087X0Q4_HUMAN	Immunoglobulin kappa variable 2-40 OS=Homo sapiens OX=9606 GN=IGKV2-40 PE=1 SV=1

131	sp|P0C0L5|CO4B_HUMAN	Complement C4-B OS=Homo sapiens OX=9606 GN=C4B PE=1 SV=2
132	sp|P01742|HV169_HUMAN	Immunoglobulin heavy variable 1-69 OS=Homo sapiens OX=9606 GN=IGHV1-69 PE=1 SV=2
133	tr|B1AHL2|B1AHL2_HUMAN	Fibulin-1 OS=Homo sapiens OX=9606 GN=FBLN1 PE=1 SV=1
134	sp|P01700|LV147_HUMAN	Immunoglobulin lambda variable 1-47 OS=Homo sapiens OX=9606 GN=IGLV1-47 PE=1 SV=2
135	sp|P01602|KV105_HUMAN	Immunoglobulin kappa variable 1-5 OS=Homo sapiens OX=9606 GN=IGKV1-5 PE=1 SV=2
136	tr|F8WF14|F8WF14_HUMAN	Carboxylic ester hydrolase OS=Homo sapiens OX=9606 GN=BCHE PE=1 SV=1
137	sp|P06312|KV401_HUMAN	Immunoglobulin kappa variable 4-1 OS=Homo sapiens OX=9606 GN=IGKV4-1 PE=1 SV=1
138	tr|B1AKG0|B1AKG0_HUMAN	Complement factor H-related protein 1 OS=Homo sapiens OX=9606 GN=CFHR1 PE=1 SV=1
139	tr|A0A0A0MRJ7|A0A0A0MRJ7_HUMAN	Coagulation factor V OS=Homo sapiens OX=9606 GN=F5 PE=1 SV=1
140	sp|P04433|KV311_HUMAN	Immunoglobulin kappa variable 3-11 OS=Homo sapiens OX=9606 GN=IGKV3-11 PE=1 SV=1
141	sp|P01772|HV333_HUMAN	Immunoglobulin heavy variable 3-33 OS=Homo sapiens OX=9606 GN=IGHV3-33 PE=1 SV=2
142	tr|E9PFZ2|E9PFZ2_HUMAN	Ceruloplasmin OS=Homo sapiens OX=9606 GN=CP PE=1 SV=1
143	sp|A0A0C4DH33|HV124_HUMAN	Immunoglobulin heavy variable 1-24 OS=Homo sapiens OX=9606 GN=IGHV1-24 PE=3 SV=1
144	tr|E9PAQ1|E9PAQ1_HUMAN	Properdin OS=Homo sapiens OX=9606 GN=CFP PE=1 SV=1
145	sp|Q14520|HABP2_HUMAN	Hyaluronan-binding protein 2 OS=Homo sapiens OX=9606 GN=HABP2 PE=1 SV=1
146	sp|P04432|KVD39_HUMAN	Immunoglobulin kappa variable 1D-39 OS=Homo sapiens OX=9606 GN=IGKV1D-39 PE=3 SV=2
147	sp|P0DP07|HV431_HUMAN	Immunoglobulin heavy variable 4-31 OS=Homo sapiens OX=9606 GN=IGHV4-31 PE=3 SV=1
148	sp|P35908|K22E_HUMAN	Keratin, type II cytoskeletal 2 epidermal OS=Homo sapiens OX=9606 GN=KRT2 PE=1 SV=2
149	sp|A0A075B6I9|LV746_HUMAN	Immunoglobulin lambda variable 7-46 OS=Homo sapiens OX=9606 GN=IGLV7-46 PE=3 SV=4
150	sp|P13645|K1C10_HUMAN	Keratin, type I cytoskeletal 10 OS=Homo sapiens OX=9606 GN=KRT10 PE=1 SV=6
151	sp|P08519|APOA_HUMAN	Apolipoprotein(a) OS=Homo sapiens OX=9606 GN=LPA PE=1 SV=1
152	sp|P01721|LV657_HUMAN	Immunoglobulin lambda variable 6-57 OS=Homo sapiens OX=9606 GN=IGLV6-57 PE=1 SV=2
153	sp|P06681|CO2_HUMAN	Complement C2 OS=Homo sapiens OX=9606 GN=C2 PE=1 SV=2
154	sp|P08571|CD14_HUMAN	Monocyte differentiation antigen CD14 OS=Homo sapiens OX=9606 GN=CD14 PE=1 SV=2
155	sp|Q9BV73|CP250_HUMAN	Centrosome-associated protein CEP250 OS=Homo sapiens OX=9606 GN=CEP250 PE=1 SV=2
156	sp|O95497|VNN1_HUMAN	Pantetheinase OS=Homo sapiens OX=9606 GN=VNN1 PE=1 SV=2
157	tr|G3V0E5|G3V0E5_HUMAN	Transferrin receptor (P90, CD71), isoform CRA_c OS=Homo sapiens OX=9606 GN=TFRC PE=1 SV=1
158	tr|Q5VVP7|Q5VVP7_HUMAN	C-reactive protein OS=Homo sapiens OX=9606 GN=CRP PE=1 SV=1
159	sp|A0A0C4DH69|KV109_HUMAN	Immunoglobulin kappa variable 1-9 OS=Homo sapiens OX=9606 GN=IGKV1-9 PE=3 SV=1
160	tr|A0A087WXI2|A0A087WXI2_HUMAN	IgGFc-binding protein (Fragment) OS=Homo sapiens OX=9606 GN=FCGBP PE=1 SV=2
161	tr|A0A024R6I7|A0A024R6I7_HUMAN	Alpha-1-antitrypsin OS=Homo sapiens OX=9606 GN=SERPINA1 PE=1 SV=1

162	sp|A0A075B6K5|LV39_HUMAN	Immunoglobulin lambda variable 3-9 OS=Homo sapiens OX=9606 GN=IGLV3-9 PE=3 SV=1
163	sp|A0A0C4DH68|KV224_HUMAN	Immunoglobulin kappa variable 2-24 OS=Homo sapiens OX=9606 GN=IGKV2-24 PE=3 SV=1
164	sp|A0M8Q6|IGLC7_HUMAN	Immunoglobulin lambda constant 7 OS=Homo sapiens OX=9606 GN=IGLC7 PE=1 SV=3
165	sp|A0A0B4J1X5|HV374_HUMAN	Immunoglobulin heavy variable 3-74 OS=Homo sapiens OX=9606 GN=IGHV3-74 PE=3 SV=1
166	tr|A0A0C4DH35|A0A0C4DH35_HUMAN	Immunoglobulin heavy variable 3-35 (non-functional) (Fragment) OS=Homo sapiens OX=9606 GN=IGHV3-35 PE=1 SV=1
167	tr|A0A0G2JMB2|A0A0G2JMB2_HUMAN	Immunoglobulin heavy constant alpha 2 (Fragment) OS=Homo sapiens OX=9606 GN=IGHA2 PE=1 SV=1
168	sp|A0A0J9YXX1|HV5X1_HUMAN	Immunoglobulin heavy variable 5-10-1 OS=Homo sapiens OX=9606 GN=IGHV5-10-1 PE=3 SV=1
169	sp|A0A075B6K4|LV310_HUMAN	Immunoglobulin lambda variable 3-10 OS=Homo sapiens OX=9606 GN=IGLV3-10 PE=3 SV=2
170	sp|P01766|HV313_HUMAN	Immunoglobulin heavy variable 3-13 OS=Homo sapiens OX=9606 GN=IGHV3-13 PE=1 SV=2
171	sp|P01743|HV146_HUMAN	Immunoglobulin heavy variable 1-46 OS=Homo sapiens OX=9606 GN=IGHV1-46 PE=1 SV=2
172	sp|A0A0C4DH31|HV118_HUMAN	Immunoglobulin heavy variable 1-18 OS=Homo sapiens OX=9606 GN=IGHV1-18 PE=3 SV=1
173	tr|A0A075B7D0|A0A075B7D0_HUMAN	Immunoglobulin heavy variable 1/OR15-1 (non-functional) (Fragment) OS=Homo sapiens OX=9606 GN=IGHV1OR15-1 PE=1 SV=1
174	tr|E9PQD6|E9PQD6_HUMAN	Serum amyloid A protein OS=Homo sapiens OX=9606 GN=SAA1 PE=1 SV=1
175	tr|A0A2Q2TTZ9|A0A2Q2TTZ9_HUMAN	Immunoglobulin kappa variable 1-33 OS=Homo sapiens OX=9606 GN=IGKV1D-33 PE=1 SV=1
176	tr|A0A075B7B8|A0A075B7B8_HUMAN	Immunoglobulin heavy variable 3/OR16-12 (non-functional) (Fragment) OS=Homo sapiens OX=9606 GN=IGHV3OR16-12 PE=1 SV=1
177	sp|A0A0C4DH34|HV428_HUMAN	Immunoglobulin heavy variable 4-28 OS=Homo sapiens OX=9606 GN=IGHV4-28 PE=3 SV=1
178	tr|H0YCQ7|H0YCQ7_HUMAN	Glutamine and serine-rich protein 1 (Fragment) OS=Homo sapiens OX=9606 GN=QSER1 PE=1 SV=1
179	tr|E5RGB5|E5RGB5_HUMAN	Bridging integrator 3 OS=Homo sapiens OX=9606 GN=BIN3 PE=4 SV=1
180	tr|C9J6K0|C9J6K0_HUMAN	Secreted phosphoprotein 24 (Fragment) OS=Homo sapiens OX=9606 GN=SPP2 PE=1 SV=1
181	tr|A0A2R8YGX3|A0A2R8YGX3_HUMAN	Tropomyosin alpha-4 chain OS=Homo sapiens OX=9606 GN=TPM4 PE=1 SV=1
182	sp|Q9HAK2|COE2_HUMAN	Transcription factor COE2 OS=Homo sapiens OX=9606 GN=EBF2 PE=2 SV=4
183	sp|P55056|APOC4_HUMAN	Apolipoprotein C-IV OS=Homo sapiens OX=9606 GN=APOC4 PE=1 SV=1
184	tr|M0QZ22|M0QZ22_HUMAN	Protein Smaug homolog 2 OS=Homo sapiens OX=9606 GN=SAMD4B PE=1 SV=1
185	tr|J3QS41|J3QS41_HUMAN	Probable helicase with zinc finger domain OS=Homo sapiens OX=9606 GN=HELZ PE=1 SV=1

186	tr|I3L4X8|I3L4X8_HUMAN	Integrin beta OS=Homo sapiens OX=9606 GN=ITGB3 PE=3 SV=1
187	tr|H0YLF3|H0YLF3_HUMAN	Beta-2-microglobulin (Fragment) OS=Homo sapiens OX=9606 GN=B2M PE=1 SV=1
188	tr|H0YF65|H0YF65_HUMAN	Fibrocystin-L (Fragment) OS=Homo sapiens OX=9606 GN=PKHD1L1 PE=1 SV=1
189	tr|H0YCW5|H0YCW5_HUMAN	DENN domain-containing protein 5A (Fragment) OS=Homo sapiens OX=9606 GN=DENND5A PE=1 SV=1
190	tr|E7ET86|E7ET86_HUMAN	Cyclin-dependent kinase-like 3 OS=Homo sapiens OX=9606 GN=CDKL3 PE=1 SV=1
191	tr|E7END6|E7END6_HUMAN	Vitamin K-dependent protein C OS=Homo sapiens OX=9606 GN=PROC PE=1 SV=1
192	tr|A0A1W2PQ80|A0A1W2PQ80_HUMAN	Immunoglobulin lambda variable 10-54 OS=Homo sapiens OX=9606 GN=IGLV10-54 PE=1 SV=1
193	sp|Q8IZC7|ZN101_HUMAN	Zinc finger protein 101 OS=Homo sapiens OX=9606 GN=ZNF101 PE=1 SV=1
194	sp|Q6BEB4|SP5_HUMAN	Transcription factor Sp5 OS=Homo sapiens OX=9606 GN=SP5 PE=2 SV=1
195	sp|Q5H9J7|BEX5_HUMAN	Protein BEX5 OS=Homo sapiens OX=9606 GN=BEX5 PE=1 SV=1
196	sp|P01833|PIGR_HUMAN	Polymeric immunoglobulin receptor OS=Homo sapiens OX=9606 GN=PIGR PE=1 SV=4
197	tr|K7ES25|K7ES25_HUMAN	Xaa-Pro dipeptidase (Fragment) OS=Homo sapiens OX=9606 GN=PEPD PE=1 SV=3
198	sp|A0A0B4J1U7|HV601_HUMAN	Immunoglobulin heavy variable 6-1 OS=Homo sapiens OX=9606 GN=IGHV6-1 PE=3 SV=1
199	sp|Q9UGM5|FETUB_HUMAN	Fetuin-B OS=Homo sapiens OX=9606 GN=FETUB PE=1 SV=2
200	sp|A0A0A0MS14|HV145_HUMAN	Immunoglobulin heavy variable 1-45 OS=Homo sapiens OX=9606 GN=IGHV1-45 PE=3 SV=1
201	sp|P33151|CADH5_HUMAN	Cadherin-5 OS=Homo sapiens OX=9606 GN=CDH5 PE=1 SV=5
202	sp|A0A0B4J1X8|HV343_HUMAN	Immunoglobulin heavy variable 3-43 OS=Homo sapiens OX=9606 GN=IGHV3-43 PE=3 SV=1
203	sp|P48740|MASP1_HUMAN	Mannan-binding lectin serine protease 1 OS=Homo sapiens OX=9606 GN=MASP1 PE=1 SV=3
204	sp|A0A0C4DH25|KVD20_HUMAN	Immunoglobulin kappa variable 3D-20 OS=Homo sapiens OX=9606 GN=IGKV3D-20 PE=3 SV=1
205	tr|A0A2R8Y7X9|A0A2R8Y7X9_HUMAN	Uncharacterized protein OS=Homo sapiens OX=9606 PE=3 SV=1
206	tr|G3V2W1|G3V2W1_HUMAN	Protein Z-dependent protease inhibitor OS=Homo sapiens OX=9606 GN=SERPINA10 PE=1 SV=1
207	sp|P01701|LV151_HUMAN	Immunoglobulin lambda variable 1-51 OS=Homo sapiens OX=9606 GN=IGLV1-51 PE=1 SV=2
208	sp|P02745|C1QA_HUMAN	Complement C1q subcomponent subunit A OS=Homo sapiens OX=9606 GN=C1QA PE=1 SV=2

Supplemental table 2. Demographic characters, frequency of specific co-morbidities, pharmacotherapy and clinical parameters of the COVID-19 patients compared between two arms of the trial.

**General**	**SOC arm**	**CPT arm**
Male	27 (67.5%)	30 (75%)
Female	13 (32.5%)	10 (25%)
Hospital admission to enrolment (days)	3.85±2.63	4.2±2.21
**Co-morbidities**	**SOC arm**	**CPT arm**
Type 2 diabetes	24 (60%)	23 (57.5%)
Hypertension	17 (42.5%)	18 (45%)
Chronic obstructive pulmonary disease	3 (7.5%)	2 (5%)
Hypothyroidism	3 (7.5%)	5 (12.5%)
Dyslipedemia	1 (2.5%)	5 (12.5%)
History of cerebrovascular disease	1 (2.5%)	1 (2.5%)
History of ischemic heart disease	2 (5%)	2 (5%)
**Pharmacotherapy**	**SOC arm**	**CPT arm**
Hydroxycholoroquin	12 (30%)	19 (47.5%)
Ivermectin	26 (65%)	27(67.5%)
Doxycyclin	21 (52.5%)	25 (62.5%)
Azithromycin	16 (40%)	19 (47.5%)
Meropenem	7 (17.5%)	9 (22.5%)
Faropenem	9 (22.5%)	3 (7.5%)
Ceftriaxone	4 (10%)	4 (10%)
Remdesivir	13 (32.5%)	12 (30%)
Intravenous corticosteroid (Dexamethasone 8mg once daily or Hydrocortisone 100mg thrice daily)	19 (47.5%)	21 (52.5%)
Oral corticosteroid (Methylprednisolone 16-20mg twice daily)	14 (35%)	9 (22.5%)
Metformin	8 (20%)	11 (27.5%)
Insulin	17 (42.5%)	17 (42.5%)
DPP4 inhibitor	0 (0%)	4 (10%)
Monteleukast	7 (17.5%)	5 (12.5%)
Prophylactic low mol wt heparin	24 (60%)	28 (70%)
Therapeutic low mol wt heparin	5 (12.5%)	4 (10%)
**Laboratory medicine parameters** (Statistics of available data)	**SOC arm**	**CPT arm**
Capillary blood glucose (mg/dl)	263±73.20082726	165.5 ± 69.85294
Random blood sugar (mg/dl)	167±91.27421572	189 ± 80.38173
Creatinine (mg/dl)	1.05±2.048229738	1.07 ± 0.429845
Urea (mg/dl)	36 ± 46.71955	36 ± 16.43402
Cholesterol (mg/dl)	164 ± 32.8527	141 ± 28.28427
Triglycerides (mg/dl)	198 ± 99.66343	140 ± 56.56854
High density lipoprotein (mg/dl)	30 ± 10.37786	40.5 ± 7.778175

Low density lipoprotein (mg/dl)	83.5 ± 222.124	90 ± 450.3847
Total Bilirubin (mg/dl)	0.77 ± 14.26836	0.73 ± 0.333992
Serum glutamic pyruvic transaminase (U/L)	43 ± 50.787	42.5 ± 32.25641
serum glutamic-oxaloacetic transaminase (U/L)	59 ± 36.31843	61 ± 30.86701
Alkaline phosphatase (U/L)	89 ± 34.42506	77 ± 51.90104
Total protein (gm/dl)	6.6 ± 0.732244	6.8 ± 0.53477
Albumin (gm/dl)	3.7 ± 0.371654	3.96 ± 0.406938
Globulin (gm/dl)	2.7 ± 0.657205	3.1 ± 0.5118
Na+ (mmol/L)	138.1 ± 7.034662	138.8 ± 6.104704
Ca2+ (mmol/L)	0.78 ± 58.78308	0.61 ± 0.273106
K+ (mmol/L)	4.18 ± 0.70784	4.28 ± 0.758733
Cl- (mmol/L)	106 ± 5.61587	102 ± 11.38294
pH	7.4775 ± 0.041449	7.482 ± 0.043903
Amylase (U/L)	55 ± 32.04684	55 ± 89.94072
Lipase (U/L)	30 ± 30.11644	46 ± 26
Hemoglobin% (gm/dl)	10.55 ± 2.072555	11.15 ± 1.669222
Red blood cells (million/mm^3)	3.91 ± 0.737673	4.21 ± 0.729966
White blood cells (per cu.mm)	8500 ± 22998.71	7850 ± 4219.75
Neutrophil (%)	78 ± 10.09578	78 ± 14.7768
Lymphocyte (%)	18 ± 9.258997	17 ± 10.1727
Monocyte (%)	2 ± 1.011759	2 ± 0.689056
Eosinophil (%)	2 ± 1.375672	2 ± 1.282245
Basophil (%)	0 ± 0	0 ± 0
Platelet (per cu.mm)	236500 ± 105176.2	164000 ± 85291.27
Packed cell volume (%)	33.5 ± 6.194703	35.65 ± 4.382482
Mean corpuscular volume (fL)	84.9 ± 19.10051	87.65 ± 5.378824
Mean corpuscular hemoglobin (pg)	26.3 ± 4.331611	27.8 ± 1.640122
Mean corpuscular haemoglobin concentration (gm/dl)	31 ± 1.50333	31.55 ± 2.748664
C Reactive Protein (mg/L)	107 ± 45.55595	105 ± 48.58326
Uric acid (mg%)	4.95 ± 2.645909	4.19 ± 1.12418

Supplemental table 3. SARS-CoV-2 Viral clades documented among the COVID-19 patients in the state of West Bengal during the time-period the trial was ongoing.

**GISAID Kolkata (331 Sequences)**		
**Clade**	**Number of sequences**	**Percentage (out of 331)**
19A	3	0.91
19B	16	4.83
20A	200	60.42
20B	88	26.59
20I/501Y.V1	22	6.65
20H/501Y.V2	2	0.60
**GISAID West Bengal (1065 Sequences)**		
**Clade**	**Number of sequences**	**Percentage (out of 1065)**
19A	34	3.19
19B	21	1.97
20A	603	56.62
20B	357	33.52
20I/501Y.V1	42	3.94
20H/501Y.V2	8	0.75
**GISAID Kolkata (September-October, 21 samples)**		
**Clade**	**Number of sequences**	**Percentage (out of 21)**
20A	16	76.19
20B	4	19.05
20I/501Y.V1	1	4.76

Supplemental table 4. Co-morbidities and final disease outcomes of individual patients randomised into two treatment arms.

No.	Co-morbidity	Treatment arm	Enrolment to discharge (days)	Enrolment to death (days)
1	Obesity, Hypothyroidism	CPT	8	NA
2	None	CPT	NA	9
3	HTN, T2DM	CPT	17	NA
4	None	CPT	19	NA
5	T2DM, HTN	CPT	6	NA
6	T2DM, HTN	CPT	NA	7
7	T2DM	CPT	8	NA
8	None	CPT	15	NA
9	HTN	CPT	NA	3
10	T2DM	CPT	NA	16
11	T2DM, HTN	CPT	10	NA
12	None	CPT	9	NA
13	T2DM, HTN	CPT	11	NA
14	T2DM, HTN, COPD	CPT	17	NA
15	T2DM, HTN	CPT	10	NA
16	T2DM, COPD	CPT	13	NA
17	HTN, H/O IHD	CPT	NA	5
18	T2DM, HTN, Hypothyroidism	CPT	20	NA
19	None	CPT	13	NA
20	None	CPT	NA	11
21	None	CPT	12	NA
22	None	CPT	8	NA
23	Aplastic anemia	CPT	10	NA
24	None	CPT	24	NA
25	HTN, T2DM	CPT	10	NA
26	T2DM	CPT	15	NA
27	None	CPT	9	NA
28	HTN, T2DM, Hypothyroidism	CPT	NA	24
29	T2DM, HTN	CPT	8	NA
30	None	CPT	17	NA
31	None	CPT	10	NA
32	None	CPT	7	NA
33	None	CPT	7	NA
34	None	CPT	25	NA
35	HTN	CPT	10	NA
36	HTN	CPT	24	NA
37	None	CPT	NA	2
38	CKD, T2DM, H/O CVA	CPT	NA	12
39	T2DM	CPT	NA	7
40	T2DM	CPT	8	NA

41	None	SOC	11	NA
42	None	SOC	13	NA
43	None	SOC	NA	1
44	None	SOC	11	NA
45	None	SOC	NA	8
46	None	SOC	10	NA
47	HTN	SOC	NA	15
48	HTN, H/O CVA	SOC	9	NA
49	None	SOC	14	NA
50	None	SOC	17	NA
51	T2DM, HTN	SOC	6	NA
52	T2DM	SOC	NA	3
53	None	SOC	25	NA
54	T2DM, CKD	SOC	21	NA
55	T2DM, Hypothyroidism	SOC	NA	7
56	COPD	SOC	28	NA
57	HTN	SOC	NA	18
58	T2DM	SOC	13	NA
59	HTN, T2DM	SOC	10	NA
60	HTN	SOC	9	NA
61	None	SOC	49	NA
62	None	SOC	NA	21
63	None	SOC	NA	16
64	HTN, T2DM	SOC	NA	3
65	HTN, T2DM, Hypothyroidism	SOC	14	NA
66	HTN	SOC	16	NA
67	HTN, ASTHMA	SOC	13	NA
68	T2DM	SOC	NA	11
69	HTN, T2DM	SOC	14	NA
70	TIA, IHD,	SOC	NA	18
71	HTN, T2DM, Hypothyroidism	SOC	NA	11
72	Takayasu Arteritis, T2DM, HTN	SOC	10	NA
73	T2DM, COPD	SOC	26	NA
74	T2DM, CKD	SOC	17	NA
75	None	SOC	17	NA
76	None	SOC	11	NA
77	None	SOC	NA	6
78	T2DM, HTN	SOC	9	NA
79	T2DM	SOC	NA	1
80	T2DM	SOC	12	NA

Supplementary note 1

Trial protocol

INTRODUCTION

INTRODUCTION

The coronavirus disease (COVID-19) or the novel coronavirus is an infectious disease that has spread globally, with more than 3 million confirmed infected cases worldwide resulting in more than 200,000 fatalities. On 11th of March, 2020, the World Health Organization (WHO) has declared COVID-19 to be a pandemic and research laboratories all over the world are working towards developing a vaccine and other therapeutic interventions.

Beyond supportive care, there are currently no proven therapeutic options for pneumonia due to coronavirus disease (COVID-19), the infection caused by Severe Acute Respiratory Syndrome Coronavirus 2 (SARS-CoV-2). Human convalescent plasma is an option for treatment of COVID-19 and will be available when sufficient numbers of people have recovered. Such persons should have high titer neutralizing immunoglobulin-containing plasma.

Passive antibody therapy involves the administration of antibodies or antibody-rich plasma against pathogens in susceptible or infected individuals for the purpose of preventing or treating disease due to that agent. In contrast, active vaccination requires the induction of an immune response that takes time to develop with responses that vary among recipients: some immunocompromised patients fail to achieve an adequate immune response. Thus, passive antibody administration is the only means of providing immediate immunity to susceptible persons and immunity of any measurable kind for highly immunocompromised patients.

Passive antibody therapy dates back to the 1890s. Prior to this, it was the only means of treating many infectious diseases before the advent of antimicrobial therapy in the 1940s (1,2). Experience from prior outbreaks with other coronaviruses, such as SARS-CoV-1 shows that convalescent plasma contains neutralizing antibodies to the virus (3). In the case of SARS-CoV-2, the anticipated mechanism of action by which passive antibody therapy would mediate protection is viral neutralization. However, other mechanisms may be possible, such as antibody dependent cellular cytotoxicity and/or phagocytosis. Convalescent serum was also used in the 2013 African Ebola epidemic. A small non-randomized study in Sierra Leone revealed a significant increase in survival among those treated with convalescent whole blood relative to those who received standard treatment (4).

Currently, the only antibody type available for immediate use is found in human convalescent plasma. As more individuals contract COVID-19 and recover, the number of potential donors will continue to increase.

For passive antibody therapy to be effective, a sufficient amount of antibody must be administered. When given to a COVID-19 susceptible person, this antibody will circulate in the blood, reach tissues and mitigate infection severity. Depending on antibody amount and composition, the protection conferred by transferred immunoglobulin can last from weeks to months.

Our aim in this study is to do a randomized control trial for convalescent plasma therapy in COVID-19, which involves administration of plasma from patients who have recovered from COVID-19 infection to moderate and severely ill patients, in order to transfer high titers of neutralizing antibodies to the recipient and improve their clinical status. Such studies are ongoing in different parts of the world with reports of positive outcome, which encourages us to

Dr. Biswanath Sharma Sarkar
M.D. (Medicine), M.B.B.S. (Cal),
CONSULTANT PHYSICIAN
Professor
Department of Medicine, WBMES

understand if it could be a possible therapeutic intervention for the Indian population. The convalescent plasma biorepository can thus be used either as a prophylactic treatment in the high-risk group and/or as a therapy in the critically ill patients.

Interferons are cytokines released by specific immune cells, known to be one of the most potent forms of defense against viral infections and the novel coronavirus propagates and multiplies by blocking these signaling pathways. On the other hand, proinflammatory cytokines like IL-6 released at a later stage of disease progression results in increased morbidity and poor prognosis in critically ill patients. Our additional aim is to study the changes in the levels of these cytokines and other immune cell markers to build a biomarker panel in order to determine which patient requires earlier hospitalization after disease detection. This will also provide insights on critical parameters that should govern selection of suitable donors as well as suitable recipients for the passive immunization using convalescent plasma in COVID-19.

Review of Literature

REVIEW OF LITERATURE

In the 21st century, there were two other epidemics with coronaviruses that were associated with high mortality, SARS-CoV-1 in 2003 and Middle Eastern Respiratory Syndrome (MERS) in 2012. In both outbreaks, the high mortality and absence of effective therapies led to the use of convalescent plasma. The largest study involved the treatment of 80 patients in Hong Kong withSARS-CoV-1(7).Those who were RT-PCR positive and seronegative for coronavirus at the time of therapy had improved prognosis.

Reports highlighting the mechanism and limitations of convalescent plasma therapy come from small case series.SARS plasma virus titers were reduced in three Taiwanesesurvivors of SARS following treatment with 500 ml of convalescent plasma(8).In South Korea, threepatients with MERS were treated with convalescent plasma, but only two or the donors were shownto have produced neutralizing antibodies(9). This second study highlights a challenge in using convalescent plasma, namely, that some who recover from viral disease may not have high titers of neutralizing antibody(10). Of note, analysis of convalescent sera from 99 MERS survivors showed that only 87 had neutralizing antibody with a geometric mean titer of 1:61. This suggests that antibody declines with time and/or that few patients make high titer responses. The plasma product we administer will be collected 14-21 days after symptom resolution and antibody titers will be assessed. Otherwise the donor will fulfill all of the standard donor criteria.

Importantly, there are reports that convalescent plasma was used for therapy of patients with COVID-19 in China during the current outbreak (http://www.xinhuanet.com/english/2020-02/28/c_138828177.htm). Although few details are available from the Chinese experience and published studies involved small numbers of patients, the available information suggests that convalescent plasma administration reduces viral load and was safe. It is also possible that other types of non-neutralizing antibodies are made that contribute to protection and recovery as described for other viral diseases(11).

Aims & Methodology

RATIONALE OF STUDY

The study on severe COVID-19 patients by passive immunization with convalescent plasma can provide

1. Data on safety and efficacy of convalescent plasma therapy
2. Data on immunological pathophysiology of the disease

OUTCOMES

PRIMARY OUTCOMES

1. To compare 'all cause' mortality
2. To identify the immune correlates for response to plasma therapy.

SECONDARY OUTCOMES

1. To compare recovery from ARDS in both groups
2. To compare time taken to negative viral RNA PCR
3. Adverse reaction to plasma therapy

WORK PLAN

Inclusion criteria:

Patients admitted with RNA PCR proven COVID-19 with severe disease (fever or suspected respiratory infection, plus one of the following; respiratory rate >30 breaths/min, severe respiratory distress, $SpO2 < 90\%$ at room air) with Mild ARDS(200 mmHg < $PaO2/FiO2 \leq 300$ mmHg, with PEEP or CPAP ≥ 5 cm $H2O$, or non-ventilated) and Moderate ARDS (100 mmHg < $PaO2/FiO2 \leq 200$ mmHg, with PEEP ≥ 5 cm $H2O$, or non-ventilated), within 5 to 10 days from initial presentation.

Exclusion criteria:

1. Pregnant or breastfeeding mothers
2. Patients with age less than 18 years
3. Admitted late after 10 days of initial presentation
4. Participating in any other clinical trial
5. Clinical status precluding infusion of blood products
6. Patients refusing consent

Sample size:

40 cases each in both arms

Study design: prospective open label randomized control trial

Study period: Study will start recruitment after ethical permission, expected from May15, 2020, till November 30, 2020 or after recruitment of the number of patients depicted, whichever is earlier.

Study participants: Patients to be recruited from the COVID hospitals designated by Govt of West Bengal

Definitions to be used:

Confirmed COVID-19 case: Naso-pharyngeal and oro-pharyngeal swab positive for SARS-Co-V 2 RNA PCR.

Cured COVID-19 case: Two consecutive SARS-Co-V 2 RNA PCR negative in Naso-pharyngeal and oro- pharyngeal swab.

Mild COVID: Patients with uncomplicated upper respiratory tract viral infection, may have non-specific symptoms such as fever, cough, sore throat, nasal congestion, malaise, headache. Patient with pneumonia and no signs of severe pneumonia are also in this group.

Severe COVID: fever or suspected respiratory infection, plus one of the following; respiratory rate >30 breaths/min, severe respiratory distress, SpO2< 90% at room air

Mild ARDS: 200 mmHg < PaO2/FiO2 ≤ 300 mmHg (with PEEP or CPAP ≥5 cm H2O, or non-ventilated)

Moderate ARDS: 100 mmHg < PaO2/FiO2 ≤200 mmHg with PEEP ≥5 cm H2O, or non-ventilated)

Severe ARDS: PaO2/FiO2 ≤ 100 mmHg with PEEP ≥5 cm H2O, or nonventilated)

When PaO2 is not available, SpO2/FiO2 ≤315 suggests ARDS (including innonventilated patients)

Ideal plasma donor: 21-28 days after viral PCR being negative and mounting antibody (IgG) response to SARS CoV-2.

Dead: Death due to any cause during study

Study plan:

Immune monitoring in COVID-19 patients:

COVID-19 patients will be tested for immunological (circulating cytokines; whole blood specific gene transcript abundance; frequency of key immune cells, viz. antibody producing cells, effector CD8 T cells) parameters. The immune parameters will be assessed 1) for all patients on day 1 of admission and then twice more after every three days, 2) for recipients on the day of plasma therapy before transfusion, then twice more every three days. This will be helpful 1) in selecting potential plasma donors among the recovered patients and 2) in gathering data to get insight on response to plasma therapies in the recruited recipients in the treatment group. Viral RNA and fecal nucleic acids will also be collected for viral RNA seq and fecal metagenomics in future.

Circulating immune cell frequency studies:

Antibody producing cells	CD19+ CD20+ CD38+ Cells (%PBMC)
CD T cell activation /exhaustion	CD3+ CD8+ CD44+ PD1+ CD38+ Cells (%PBMC)
Dendritic cell phenotype	Dendritic cell subsets (BDCA1+/BDCA2+/BDCA3+) (%PBMC)

Multiplex cytokine assay on serum for : G-CSF, GM-CSF, IFN-α, IFN-β, IFN-γ, IL-1α, IL-1β, IL-2, IL-3, IL-4, IL-5, IL-6, IL-9, IL-10, IL-12 (p40), IL-12 (p70), IL-13, IL-15, IL-16, IL-17A, IL-17F, IL-18, IL-21, IL-22, IL-23p19, IL-25, IL-27p28, IL-28A, IL-31, KC, MCP-1 (MCAF), MIP-1α, MIP-1β, RANTES, TNF-α, FGF Basic, M-CSF, MIG, MIP-2, PDGF-BB, VEGF, CTACK / CCL27, Eotaxin / CCL11, IP-10 / CXCL10, MCP-3 / CCL7, SDF-1α / CXCL12.

Recovered patients (Convalescent plasma donors):

Plasmapheresis: (Ref: File. X- 11026/78/2020-BD, CDSCO, DGHS, GOI dated 17.04.202020 entitled: Clinical trial in convalescent plasma in COVID-19 patients)

Donor inclusion criteria:
Recovered COVID-19 male patients and nulliparous females only (at 28 days convalescence period from last PCR negative or two non reactive RT-PCR results of nasopharyngel swabs taken within 24 hrs if the procedure has to be performed earlier), aged 18-50 years, with bodyweight >55kg, Hemoglobin/Hematocrit: 12.5 / 38%, Platelet count: >1,50,000/ul, normal plasma albumin level and serum protein level above 6 gm/dl in case of repeat donation, favorable peripheral venous access, on provision of informed consent from donor.

Donor exclusion criteria:
Any of the donors having any other comorbid conditions like cancer, chronic kidney disease, chronic infectious diseases, Hypertension with systemic organ failure, persons on chronic steroid regime, diabetes mellitus with multisystem involvement, abnormally high plasma albumin level as well as all deferral criteria as per Drugs and Cosmetics Act and its ammendments and recent NBTC guidelines of blood donor deferral .

Pre-procedure test:

Complete Blood Count (Hb%, Platelet) ABO-RH, Antibody screen, mandatory TTI screen (HIV, HBsAg, HCV,MP and syphilis by serology and NAT). Serum protein (albumin) approximately 10 ml EDTA and 5 ml Clotted blood sample should be drawn

Plasmapheresis procedure:

Instrument: MCS Plus (Haemonetics Corp., Braintree, USA)
Proposed plasma volume collection: 500 ml at a time at not more than 1000 ml in a fortnight if procedure is repeated

Processing and storage: The plasma will be kept in 200-250ml aliquots under sterile connection and cryostored quarantine freezer at -80 deg C, with proper label and samples, unless used immediately. The record of each individual unit shall be kept in separate register.

Precounselling: Precounselling and selection of potential donors will be done beforehand. Donors will be advised to have a light breakfast on the day of procedure. Transportation of the donors will be arranged by the study group.

Post-donation: Post donation, each donor will be observed for an hour before transportation back home.

Management of adverse events
Any adverse event related to plasmapheresis is notified managed as per departmental SOP and under strict medical supervision

Any adverse events related to the patient transfusion has to be managed by the treating facility and notified to the Dept. IHBT for the course of action

COVID-19 patients (Potential RCT recruitments):

COVID-19 patients on provision of informed consent, after assessment as per the inclusion criteria, will be randomized into two arms. One experimental group will get plasma (200ml, OD, two consecutive days) in addition to SOC (Standard of care) therapy and the control group will get SOC therapy only. Both the group will be observed till the end of therapy.

Both the groups will also be tested for immunological (circulating cytokines; whole blood specific gene transcript abundance; frequency of key immune cells, viz. antibody producing cells, effector CD8 T cells) and hormonal profiles like serum cortisol and ACTH from blood. Nasopharyngeal and oropharyngeal swab and stool samples of the patients will be preserved for sequencing studies.

Randomization:

Patients enrolled in this study will be randomized using an online randomization tool in order to get recruited in the plasma therapy group at a 1:1 ratio. The process of randomization will be stratified by the risk status: high risk and normal risk as defined by ICMR.

Sample size and power calculation:

Dr. Soumyanath Sharma Sarkar
M.D. (Medicine), M.B.B.S. (Cal),
CONSULTANT PHYSICIAN
Professor
Department of Medicine, WBMES

This study plans a sample size of 80 subjects, which will be randomized in a 1:1 ratio to the group receiving COVID-19 convalescent plasma vs control group.
We calculated the power of the study with following assumptions:
The analysis will compare the efficacy in the convalescent titer plasma and control groups using proportional odds model and a two-sided Type-I error rate (alpha) of 0.05 and Type-II error rate (beta) of 0.2. The incidence of symptomatic disease of 28% in exposed patients treated with SOC only whereas, 5% for patients treated with convalescent plasma added to SOC.
We estimated a sample size of 80 patients (40 in each arm) would be sufficient in order to detect a difference in outcomes between two arms with power of 0.8.

Duration and dosage of convalescent plasma therapy:

200ml COVID-19 plasma will be transfused once daily on two consecutive days in patients in the experimental group.

Follow up plan: Both the group after discharge will be followed up till 28 days after discharge.

Outcome parameters:

1. Progression to severe ARDS and recovery from severe ARDS
2. Immune correlates of response to plasma therpay
3. 'All cause' mortality
4. Time to become PCR negative from initial presentation
5. Incidence of minor and major transfusion reactions

Statistical analysis:

Appropriate statistical methods shall be employed to compare the major outcome variables. We will estimate the cumulative incidence for each arm using a non-parametric estimator (e.g. Kaplan-Meier). The adjustment of the baseline covariates will be done using inverse probability of treatment weights in order to increase the power of the trial. The adjustments will be done for factors like age, being immunocompromised and presence of additional comorbidities. Chi-square test shall be used for the Qualitative variables. Students T-test and non-parametric tests shall be used for the quantitative variables. Finally, we will use a two-sided Type I error rate of 0.05 and 95% CI for statistical inference. Furthermore, an adverse event (AE) data analysis will be descriptive based on MedDRA coding of events. AE will be compared between randomized arms using Fisher's exact Test.

REFERENCES

1. Casadevall A, Scharff MD. Return to the past: the case for antibody-based therapies in infectious diseases. Clinical infectious diseases. 1995;21(1):150–161.

2. Casadevall A, Dadachova E, Pirofski L. Passive antibody therapy for infectious diseases. Nature Reviews Microbiology. 2004;2(9):695–703.

3. Zhang J-S, Chen J-T, Liu Y-X, Zhang Z-S, Gao H, Liu Y, et al. A serological survey on neutralizing antibody titer of SARS convalescent sera. Journal of medical virology. 2005;77(2):147–150.

4. Sahr F, Ansumana R, Massaquoi T, Idriss B, Sesay F, Lamin J, et al. Evaluation of convalescent whole blood for treating Ebola Virus Disease in Freetown, Sierra Leone. Journal of Infection. 2017;74(3):302–309.

5. Casadevall A, Pirofski L. Antibody-mediated regulation of cellular immunity and the inflammatory response. TRENDS in Immunology. 2003;24(9):474–478.

6. Casadevall A, Scharff MD. Serum therapy revisited: animal models of infection and development of passive antibody therapy. Antimicrobial agents and chemotherapy. 1994;38(8):1695.

7. Cheng Y, Wong R, Soo Y, Wong W, Lee C, Ng M, et al. Use of convalescent plasma therapy in SARS patients in Hong Kong. European Journal of Clinical Microbiology and Infectious Diseases. 2005;24(1):44–46.

8. Yeh K-M, Chiueh T-S, Siu L, Lin J-C, Chan PK, Peng M-Y, et al. Experience of using convalescent plasma for severe acute respiratory syndrome among healthcare workers in a Taiwan hospital. Journal of Antimicrobial Chemotherapy. 2005;56(5):919–922.

9. Ko J-H, Seok H, Cho SY, Ha YE, Baek JY, Kim SH, et al. Challenges of convalescent plasma infusion therapy in Middle East respiratory coronavirus infection: a single centre experience. Antiviral therapy. 2018;23(7):617–622.

10. Arabi YM, Hajeer AH, Luke T, Raviprakash K, Balkhy H, Johani S, et al. Feasibility of using convalescent plasma immunotherapy for MERS-CoV infection, Saudi Arabia. Emerging infectious diseases. 2016;22(9):1554.

11. Van Erp EA, Luytjes W, Ferwerda G, Van Kasteren PB. Fc-Mediated Antibody Effector Functions During Respiratory Syncytial Virus Infection and Disease. Frontiers in immunology. 2019;10.

12. Wan Y, Shang J, Sun S, Tai W, Chen J, Geng Q, et al. Molecular mechanism for antibody-dependent enhancement of coronavirus entry. Journal of Virology. 2020;94(5).

13. Mair-Jenkins J, Saavedra-Campos M, Baillie JK, Cleary P, Khaw F-M, Lim WS, et al. The effectiveness of convalescent plasma and hyperimmune immunoglobulin for the treatment of severe acute respiratory infections of viral etiology: a systematic review and exploratory meta-analysis. The Journal of infectious diseases. 2015;211(1):80–90.

14. Crowe JE, Firestone C-Y, Murphy BR. Passively acquired antibodies suppress humoral but not cell-mediated immunity in mice immunized with live attenuated respiratory syncytial virus vaccines. The Journal of Immunology. 2001;167(7):3910–3918.

Dr. Biswanath Sharma Sarkar
M.D.(Medicine),M.B.B.S.(Cal),
CONSULTANT PHYSICIAN
Professor
Department of Medicine, [illegible]

Supplementary note 2

CONSORT Checklist

CONSORT 2010 checklist of information to include when reporting a randomised trial*

Section/Topic	Item No	Checklist item	Reported on page No
**Title and abstract**			
	1a	Identification as a randomised trial in the title	1
	1b	Structured summary of trial design, methods, results, and conclusions (for specific guidance see CONSORT for abstracts)	3
**Introduction**			
Background and objectives	2a	Scientific background and explanation of rationale	4
	2b	Specific objectives or hypotheses	5
**Methods**			
Trial design	3a	Description of trial design (such as parallel, factorial) including allocation ratio	6, 13
	3b	Important changes to methods after trial commencement (such as eligibility criteria), with reasons	9
Participants	4a	Eligibility criteria for participants	6
	4b	Settings and locations where the data were collected	6
Interventions	5	The interventions for each group with sufficient details to allow replication, including how and when they were actually administered	6
Outcomes	6a	Completely defined pre-specified primary and secondary outcome measures, including how and when they were assessed	8
	6b	Any changes to trial outcomes after the trial commenced, with reasons	9
Sample size	7a	How sample size was determined	13
	7b	When applicable, explanation of any interim analyses and stopping guidelines	NA
Randomisation:			
Sequence generation	8a	Method used to generate the random allocation sequence	14
	8b	Type of randomisation; details of any restriction (such as blocking and block size)	14
Allocation concealment mechanism	9	Mechanism used to implement the random allocation sequence (such as sequentially numbered containers), describing any steps taken to conceal the sequence until interventions were assigned	14
Implementation	10	Who generated the random allocation sequence, who enrolled participants, and who assigned participants to interventions	10, 14
Blinding	11a	If done, who was blinded after assignment to interventions (for example, participants, care providers, those	14

		assessing outcomes) and how	
	11b	If relevant, description of the similarity of interventions	NA
Statistical methods	12a	Statistical methods used to compare groups for primary and secondary outcomes	8, 20
	12b	Methods for additional analyses, such as subgroup analyses and adjusted analyses	8
**Results**			
Participant flow (a diagram is strongly recommended)	13a	For each group, the numbers of participants who were randomly assigned, received intended treatment, and were analysed for the primary outcome	6
	13b	For each group, losses and exclusions after randomisation, together with reasons	NA
Recruitment	14a	Dates defining the periods of recruitment and follow-up	6
	14b	Why the trial ended or was stopped	NA
Baseline data	15	A table showing baseline demographic and clinical characteristics for each group	Fig 3, Suppl table 2
Numbers analysed	16	For each group, number of participants (denominator) included in each analysis and whether the analysis was by original assigned groups	6
Outcomes and estimation	17a	For each primary and secondary outcome, results for each group, and the estimated effect size and its precision (such as 95% confidence interval)	6
	17b	For binary outcomes, presentation of both absolute and relative effect sizes is recommended	NA
Ancillary analyses	18	Results of any other analyses performed, including subgroup analyses and adjusted analyses, distinguishing pre-specified from exploratory	NA
Harms	19	All important harms or unintended effects in each group (for specific guidance see CONSORT for harms)	9
**Discussion**			
Limitations	20	Trial limitations, addressing sources of potential bias, imprecision, and, if relevant, multiplicity of analyses	10
Generalisability	21	Generalisability (external validity, applicability) of the trial findings	10
Interpretation	22	Interpretation consistent with results, balancing benefits and harms, and considering other relevant evidence	10
**Other information**			
Registration	23	Registration number and name of trial registry	3, 12
Protocol	24	Where the full trial protocol can be accessed, if available	Suppl note 1
Funding	25	Sources of funding and other support (such as supply of drugs), role of funders	10

*We strongly recommend reading this statement in conjunction with the CONSORT 2010 Explanation and Elaboration for important clarifications on all the items. If relevant, we also recommend reading CONSORT extensions for cluster randomised trials, non-inferiority and equivalence trials, non-pharmacological treatments, herbal interventions, and pragmatic trials. Additional extensions are forthcoming: for those and for up to date references relevant to this checklist, see www.consort-statement.org.
