# Supplementary material for: A phase 2 single center open label randomised control trial for convalescent plasma therapy in patients with severe COVID-19
Source: Nat Commun. 2022 Jan 19;13:383. doi: 10.1038/s41467-022-28064-7 (PMC8770561; doi:10.1038/s41467-022-28064-7)
Supplement: Supplementary file 3 — Reporting Summary [file 41467_2022_28064_MOESM3_ESM.pdf]

nature research

Corresponding author(s): Yogiraj Ray, Dipyaman Ganguly

Last updated by author(s): Dec 7, 2021

Reporting Summary

Nature Research wishes to improve the reproducibility of the work that we publish. This form provides structure for consistency and transparency in reporting. For further information on Nature Research policies, see our Editorial Policies and the Editorial Policy Checklist.

Statistics

For all statistical analyses, confirm that the following items are present in the figure legend, table legend, main text, or Methods section.

n/a	Confirmed	
☐	☒	The exact sample size (n) for each experimental group/condition, given as a discrete number and unit of measurement
☐	☒	A statement on whether measurements were taken from distinct samples or whether the same sample was measured repeatedly
☐	☒	The statistical test(s) used AND whether they are one- or two-sided<br>*Only common tests should be described solely by name; describe more complex techniques in the Methods section.*
☐	☒	A description of all covariates tested
☐	☒	A description of any assumptions or corrections, such as tests of normality and adjustment for multiple comparisons
☐	☒	A full description of the statistical parameters including central tendency (e.g. means) or other basic estimates (e.g. regression coefficient) AND variation (e.g. standard deviation) or associated estimates of uncertainty (e.g. confidence intervals)
☐	☒	For null hypothesis testing, the test statistic (e.g. F, t, r) with confidence intervals, effect sizes, degrees of freedom and P value noted<br>*Give P values as exact values whenever suitable.*
☒	☐	For Bayesian analysis, information on the choice of priors and Markov chain Monte Carlo settings
☒	☐	For hierarchical and complex designs, identification of the appropriate level for tests and full reporting of outcomes
☐	☒	Estimates of effect sizes (e.g. Cohen's d, Pearson's r), indicating how they were calculated

Our web collection on statistics for biologists contains articles on many of the points above.

Software and code

Policy information about availability of computer code

Data collection	N/A
Data analysis	N/A

For manuscripts utilizing custom algorithms or software that are central to the research but not yet described in published literature, software must be made available to editors and reviewers. We strongly encourage code deposition in a community repository (e.g. GitHub). See the Nature Research guidelines for submitting code & software for further information.

Data

Policy information about availability of data

All manuscripts must include a data availability statement. This statement should provide the following information, where applicable:

- Accession codes, unique identifiers, or web links for publicly available datasets
- A list of figures that have associated raw data
- A description of any restrictions on data availability

Data availability statement complies with the requirements.

Field-specific reporting

Life sciences study design

All studies must disclose on these points even when the disclosure is negative.

Sample size	A sample size calculation was performed with incidence of death among COVID-19 patients suffering from ARDS assumed to be 28% in the standard-of-care group based on data of preceding two months (March and April, 2020) from the clinical trial site. The incidence of death in the CPT-treated group was anticipated to be reduced to 5%. A sample size of 40 in each group was calculated taking alpha = 0.05 and power to be 80%. Based on this calculation a sample size of 40 in each group was decided upon and sought approval from the regulatory authorities.
Data exclusions	N/A
Replication	N/A
Randomization	A computer-generated random sequence generated by Dr. Sandip Paul (CSIR-IICB) were conveyed to the responsible clinicians (Dr. Yogiraj ray ad Dr. Shekhar Ranjan Paul) for randomised assignment of patients into two arms as and when they met the inclusion criteria.
Blinding	It was an open label RCT. Randomised assignments to each arm could not be blinded due to logistic issues around patient recruitments and sample collections in COVID-19 isolation wards.

Reporting for specific materials, systems and methods

We require information from authors about some types of materials, experimental systems and methods used in many studies. Here, indicate whether each material, system or method listed is relevant to your study. If you are not sure if a list item applies to your research, read the appropriate section before selecting a response.

Materials & experimental systems

n/a	Involved in the study	
☒	☐	Antibodies
☒	☐	Eukaryotic cell lines
☒	☐	Palaeontology and archaeology
☒	☐	Animals and other organisms
☐	☒	Human research participants
☐	☒	Clinical data
☒	☐	Dual use research of concern

Methods

n/a	Involved in the study	
☒	☐	ChIP-seq
☒	☐	Flow cytometry
☒	☐	MRI-based neuroimaging

Human research participants

Policy information about studies involving human research participants

Population characteristics	Convalescent donors:<br>61 convalescent individuals (female: N=12, Age: 26 ± 2.98 years; male: N=49, Age: 35.37 ± 9.06 years) who recovered from COVID-19 at least 28 days prior to screening, were screened for eligibility for plasma donation. 46 donors were found eligible for plasmapheresis. All of them were screened at 40-80 days after they were first tested positive on RT-PCR for SARS-CoV-2.<br><br>COVID-19 recipients:<br>80 patients, fulfilling the inclusion criteria were recruited into the trial and randomized into either standard of care (SOC) arm or the convalescent plasma therapy (CPT) arm. Demographic characteristics of the patients between two parallel arms were not significantly different [Male: 27 (SOC), 30 (CPT); Female: 13 (SOC), 10 (CPT); Hospital admission to enrolment (days): 3.85 ±2.63 (SOC); 4.2±2.21 (CPT)]
Recruitment	A computer-generated random sequence generated by Dr. Sandip Paul (CSIR-IICB) were conveyed to the responsible clinicians (Dr. Yogiraj ray ad Dr. Shekhar Ranjan Paul) for randomised assignment of patients into two arms as and when they met the inclusion criteria. No selection bias was noted.
Ethics oversight	The randomized control trial (RCT) on passive immunization with convalescent plasma therapy and all associated studies were done with informed consent from the patients according to the recommendations and ethical approval from the Institutional Review Boards of all the concerned institutions, viz. CSIR-Indian Institute of Chemical Biology, Kolkata, India (IICB/IRB/2020/3P), Medical College Hospital, Kolkata (MC/KOL/IEC/NON-SPON/710/04/2020), India and Infectious Disease & Beleghata General Hospital (ID & BG Hospital), Kolkata, India (IDBGH/Ethics/2429). The RCT was approved by Central Drugs Standard Control Organisation (CDSCO) under Directorate General of Health Services, Ministry of Health & Family Welfare, Govt. of India (approval no. CT/BP/09/2020) and registered with Clinical Trial Registry of India (CTRI, No. CTRI/2020/05/025209), under Indian Council of Medical Research, India.

Note that full information on the approval of the study protocol must also be provided in the manuscript.

Clinical data

Policy information about clinical studies

All manuscripts should comply with the ICMJEguidelines for publication of clinical research and a completedCONSORT checklist must be included with all submissions.

Clinical trial registration	CTRI/2020/05/025209
Study protocol	Available with the authors. Explained in the manuscript.
Data collection	Patients (recipients) were recruited between May 31, 2020 and October 12, 2020. Clinical data were collected from the hospital.
Outcomes	PRIMARY OUTCOMES<br>1. To compare 'all cause' mortality (30 days)<br>2. To identify the immune correlates for response to plasma therapy.<br><br>SECONDARY OUTCOMES<br>1. To compare recovery from ARDS in both groups<br>2. To compare time taken to negative viral RNA PCR<br>3. Adverse reaction to plasma therapy<br><br>Secondary outcome no.2 could not be fulfilled as the standard protocol prescribed by Indian Council of Medical Research for COVID-19 management excluded the necessity for negative RT-PCR before hospital discharge. The inability to gather data for the secondary outcome 2 was informed to the institutional IRB.<br>The secondary outcome 1 on time taken for recovery from ARDS in all patients could not be determined accurately for all patients due to emergent operational limitations in access to computed tomography facility and arterial blood gas analysis for follow-up. Instead, recovery from COVID-19 disease was assessed in terms of time taken for discharge from hospital, though it was not pre-specified in the trial protocol. This was also informed to the institutional IRB.
